# Synthetic methylotrophic yeasts for the sustainable fuel and chemical production

**DOI:** 10.1186/s13068-022-02210-1

**Published:** 2022-10-22

**Authors:** Vanessa Wegat, Jonathan T. Fabarius, Volker Sieber

**Affiliations:** 1 Fraunhofer Institute for Interfacial Engineering and Biotechnology, Straubing branch Biocat, Schulgasse 11a, 94315 Straubing, Germany; 2 Technical University of Munich, Campus Straubing for Biotechnology and Sustainability, Schulgasse 16, 94315 Straubing, Germany

**Keywords:** Methylotrophy, Yeasts, Bioeconomy, Biofuels, Synthetic metabolism, Non-traditional feedstock, Bacteria

## Abstract

Global energy-related emissions, in particular carbon dioxide, are rapidly increasing. Without immediate and strong reductions across all sectors, limiting global warming to 1.5 °C and thus mitigating climate change is beyond reach. In addition to the expansion of renewable energies and the increase in energy efficiency, the so-called Carbon Capture and Utilization technologies represent an innovative approach for closing the carbon cycle and establishing a circular economy. One option is to combine CO_2_ capture with microbial C_1_ fermentation. C_1_-molecules, such as methanol or formate are considered as attractive alternative feedstock for biotechnological processes due to their sustainable production using only CO_2_, water and renewable energy. Native methylotrophic microorganisms can utilize these feedstock for the production of value-added compounds. Currently, constraints exist regarding the understanding of methylotrophic metabolism and the available genetic engineering tools are limited. For this reason, the development of synthetic methylotrophic cell factories based on the integration of natural or artificial methanol assimilation pathways in biotechnologically relevant microorganisms is receiving special attention. Yeasts like *Saccharomyces cerevisiae* and *Yarrowia lipolytica* are capable of producing important products from sugar-based feedstock and the switch to produce these in the future from methanol is important in order to realize a CO_2_-based economy that is independent from land use. Here, we review historical biotechnological applications, the metabolism and the characteristics of methylotrophic yeasts. Various studies demonstrated the production of a broad set of promising products from fine chemicals to bulk chemicals by applying methylotrophic yeasts. Regarding synthetic methylotrophy, the deep understanding of the methylotrophic metabolism serves as the basis for microbial strain engineering and paves the way towards a CO_2_-based circular bioeconomy. We highlight design aspects of synthetic methylotrophy and discuss the resulting chances and challenges using non-conventional yeasts as host organisms. We conclude that the road towards synthetic methylotrophic yeasts can only be achieved through a combination of methods (e.g., metabolic engineering and adaptive laboratory evolution). Furthermore, we presume that the installation of metabolic regeneration cycles such as supporting carbon re-entry towards the pentose phosphate pathway from C_1_-metabolism is a pivotal target for synthetic methylotrophy.

## Background

Encountering climate change and mitigating its impact on the environment, on our economies and on the society is the defining challenge of our time. Due to the rapid growth of the world's population, the demand for energy is increasing dramatically every year and CO_2_-neutral solutions are desperately needed. The use of conventional energy sources (e.g., oil, coal and natural gas) represents by far the largest source of greenhouse gas emissions from human activities and thus contributes significantly to global warming. The depletion of fossil fuels and historical and on-going geopolitical conflicts are further reasons to commit to renewable energy sources.

In this regard, the capture of CO_2_ from an (industrial) process or even directly from the air and its subsequent utilization (Carbon Capture and Utilization, CCU) is one option to reduce industrial emissions and realizing a circular economy, provided that the energy used in capturing and converting the CO_2_ is zero carbon [1]. In general, CCU refers to the capture, transport and use of carbon compounds such as carbon monoxide or carbon dioxide, in which the carbon is fed into at least one further utilization cycle. Depending on the origin and usage of the carbon, this requires the combination of different processes, each of which is associated with energy or resource consumption as well as environmental impacts. Often, gaseous CO_2_ is used, which can be of various origins (from fossil energy sources, industrial processes or raw materials, e.g., limestone) or directly from the atmosphere.

CCU is currently the most cost-effective alternative for reducing emissions from the production of bulk chemicals [2]. The predicted costs for CCU-equipped natural gas-based ammonia and methanol production are about 20–40% higher compared to their conventional production. Nevertheless, cost-reductions for CCU have already been achieved and this trend will continue as the industry proceeds with the integration of CCU. However, most significantly the CO_2_ source influences CCU costs dramatically when comparing “pure” (i.e., ethanol production or natural gas processing) or “diluted” streams (i.e., cement production and power generation) [3]. One innovative option combines the conversion of CO_2_ into C_1_ compounds like methanol or formic acid with subsequent application in microbial methylotrophic fermentation.

Methylotrophic microbes are a divergent group of microorganisms, such as bacteria or yeasts, which can harness reduced one-carbon compounds for growth, energy generation and consequently the production of value-added chemicals, materials or food and feed ingredients. In general, methanol or formate are the carbon sources of choice in terms of methylotrophy. In the context of one-carbon substrates, also the valorization of H_2_/CO_2_ and a mixture of H_2_ and CO in advanced microbial gas-fermentation aroused attention [4]. However, in biotechnology and fermentation processes, liquid C_1_ substrates (i.e., dissolved formate, methanol) support striking advantages in comparison to gaseous C_1_ substrates (i.e., CO_2_, CO). In detail, storage or handling of liquids is convenient and easy compared to gases. Even more, from the view point of the bioprocess, the feeding of gaseous substrates in the fermentation broth comes along with specific drawbacks, in terms of mass transport and gas water solubility [5]. Therefore, the feeding of highly concentrated aqueous substrate solutions in fed-batch fermentations enables efficient and controllable substrate supply.

Recently, methanol and formate received also attention due to their easy and efficient production via heterogeneous chemical catalysis or electrochemical CO_2_ reduction, respectively [6]. A CO_2_-dependent methanol production is climate-friendly and independent of fossil resource usage and consequently increases the environmental benefit while reducing CO_2_ emissions. When considering future trends in energy supply and demand, it is important to acknowledge that compelling market factors will continue to strongly influence the price of energy. Fossil fuels are not only the cause of environmental pollution and climate crisis, but also of historical and on-going conflicts and it is anticipated that future prices will increase [7]. The production of methanol by chemical or electrochemical reduction of carbon dioxide is therefore becoming gradually attractive and enables eventually the valorization of CO_2_ as an indirect fermentation substrate.

Here, we first briefly review uses and characteristics of methylotrophic yeasts in bioprocessing and their metabolism. Towards the exploitation of (methylotrophic) yeasts for the production of biofuels and other bioproducts, an overview of demonstration examples is given. In addition, the growing field of synthetic biology leads to new opportunities like the installation of synthetic methylotrophy in established microbial hosts. Hence, a deep understanding of the methylotrophic metabolism serves as the basis for synthetic methylotrophy, which can be applied to establish a sustainable CO_2_-based bioeconomy using tailor-made methylotrophic cell factories.

Recent approaches have focused on engineering synthetic methylotrophy in bacteria, such as *Escherichia coli* and *Corynebacterium glutamicum*, which have been to date harnessed for the production of various relevant chemicals. Nonetheless, yeast species like *S. cerevisiae* or non-conventional yeasts like *Y. lipolytica* also have potential as hosts for engineering synthetic methylotrophy as they provide distinct advantages over organisms such as *E. coli* for use in industrial fermentation. It was shown that protein expression is superior in terms of gene expression, protein folding, and post-translational modifications of numerous eukaryotic proteins [8, 9]. One of the most striking characteristics of yeasts is the enhanced tolerance towards acidic pH conditions [10]. Furthermore, eukaryotes are not affected by phage contamination [11]. Moreover, they possess organelles that can be used for organelle directed gene expression to harness beneficial cellular functions, for example to separate formaldehyde detoxification from the cytosol [12]. This review addresses the current state of constructing synthetic methylotrophic pathways in yeasts and how these techniques can be applied to efficiently produce ethanol, fatty acids or other industrially relevant products.

## Current state of methylotrophic yeasts in biotechnology

**Fig. 1:**
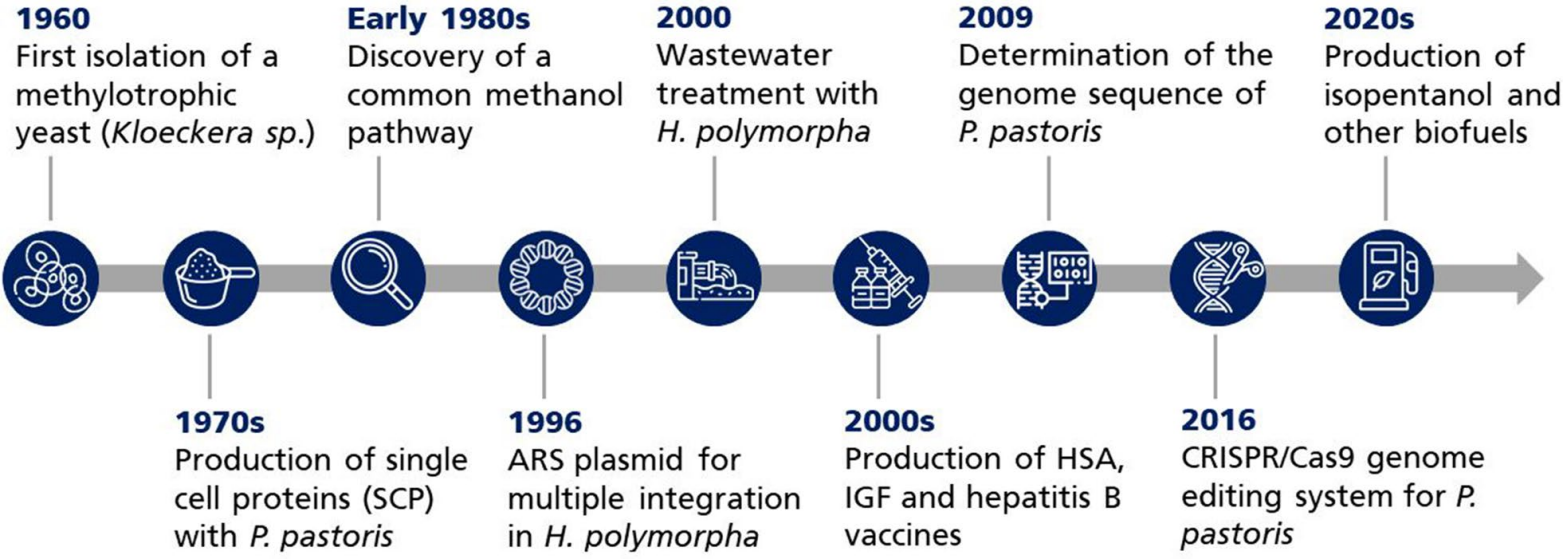
History of methylotrophic yeast research in biotechnology. Key steps during the last decades in on-going scientific efforts to understand, engineer, and develop methylotrophic eukaryotic microorganisms

Bacterial methylotrophs belong to diverse phylae, whereas eukaryotic methylotrophs are restricted to a limited number of yeast genera, including *Candida, Pichia, Ogataea, Komagataella* and *Kuraishia*. A fundamental discovery was the identification of a methanol-utilizing pathway common for all methylotrophic yeasts. While bacteria conduct the initial step either using a pyrroloquinoline quinone (PQQ)- or nicotinamide adenine dinucleotide (NAD^+^)-dependent dehydrogenase, methylotrophic yeasts harness an unspecific alcohol oxidase (AOX) using molecular oxygen as the electron acceptor [13]. Since the history of methylotrophic yeasts (Fig. 1) is already elaborated elsewhere [14, 15], we refer the reader to these reviews for a comprehensive overview and focus specifically on the genetic tools used to engineer methylotrophic yeasts and their current application in biotechnology.

### Genetic tools for the engineering of methylotrophic yeasts

In the 1980s and 1990s, numerous genetic tools to engineer methylotrophic yeasts became available, which were refined ever since. In particular, the exploration of various transformation methods [16, 17] and the design of effective vectors [18, 19] led to the production of various recombinant proteins and fine chemicals. These achievements were supported by the identification of strong methanol-inducible promoters to drive gene expression. Predominantly, the promoter of the alcohol oxidase I (AOX1) from *Pichia pastoris*, and corresponding promoters from other methylotrophic yeasts, are used for recombinant protein production [20]. Alternatively, promoters such as the *P. pastoris* GAP, FLD1, PEX8, and YPT1 promoters are used [21], whereas in *Hansenula polymorpha* the formate dehydrogenase (FMD) promoter is commonly harnessed [22].

Besides engineering the transcription initiation, less effort was laid on transcription termination. Genetic switches like transcription terminators (TT) are additionally used to adjust gene expression. Usually, the AOX1-TT and the *S. cerevisiae* derived CYC1-TT are utilized. Prielhofer et al. assessed the efficiency of different transcription promoters and terminators of strongly expressed *P. pastoris* genes. The promoter and terminator strength potential was characterized by expressing the intracellular reporter eGFP. In total, 10 terminators were tested with the GAP promoter and normalized to termination with ScCYC1-TT. Seven transcription terminator sequences resulted in a slightly higher eGFP expression compared to the widely used ScCYC1-TT [23].

Lately, the effect of six promoters and 15 terminators on fine-tuning gene expression in *H. polymorpha* was explored. The authors monitored GFP expression in batch cultivations on glucose, glycerol, and methanol or mixtures of these. Through terminator variation, a sixfold difference in gene expression was accomplished with the methanol oxidase (MOX) terminator. Using the MOX terminator resulted in around 50% higher gene expression on all carbon sources compared to the second-strongest terminator [24]. Since transcription terminators seem functional across differing yeast species, the mentioned terminators can also find application in synthetic methylotrophic yeasts [25].

A remarkable knowledge gain was achieved in 2009 and 2013, respectively, by publication of the genome sequences of *P. pastoris* [26] and *H. polymorpha* [27]. Recombinant protein production is generally achieved using integrative vectors. Nevertheless, also episomal plasmids can provide a powerful tool to accelerate cloning and high-throughput screening, which is indispensable for synthetic biology approaches. Through the identification of heterologous and endogenous autonomously replicating sequences (ARS) of *P. pastoris* by genome mining, various efficient episomal expression plasmids could be constructed [28–30]. Also for other methylotrophic yeasts, such as *H. polymorpha,* plasmids containing an autonomously replicating sequence (HARS) derived from subtelomeric regions exist [31].

Another recent groundbreaking event was the development of a CRISPR/Cas9 genome editing system for *P. pastoris*. The latter enables genetic engineering via nonhomologous end joining (NHEJ) at an outstanding high efficiency [32]. In a subsequent study, a CRISPR-based synthetic biology toolkit for the chromosomal integration and assembly of multigene biosynthetic pathways in *P. pastoris* was developed, which enabled single-locus (~ 100%), two-loci (~ 93%), and three-loci (~ 75%) integration at high efficiencies [33]. In addition, CRISPR/Cas9-mediated engineering tools were implemented for *H. polymorpha* [34]. Recently, a recombination machinery engineering was developed for enhancing homologous recombination (HR) activity together with expression of an efficient CRISPR/Cas9 system. Overexpression of proteins related to HR and downregulation of NHEJ increased HR rates up to 70%, simplifying genetic engineering in this non-conventional yeast [35].

### Application of engineered methylotrophic yeasts in biotechnology

The availability of functional genetic tools led to numerous biotechnological applications of methylotrophic yeasts. Several products were obtained using methanol or mixtures of methanol with renewable feedstock. In particular, the production of (I) human serum albumin (HSA) [36], (II) the insulin like growth factor (IGF) [37] or (III) hepatitis B vaccines [38] was achieved. Furthermore, *P. pastoris* was engineered for the production of various protein-based polymers such as (IV) collagen [39, 40], (V) gelatins [41], (VI) silk-like proteins [42, 43] and (VII) elastin-like proteins [44, 45]. Nevertheless, challenges like low yields, proteolytic degradation, and potential self-assembly in vivo may be faced when using *P. pastoris* for polymer production [46].

In a recent study, recombinant *P. pastoris* was constructed for malic acid production solely from methanol by redistribution of metabolic fluxes and deletion of genes related to by-product formation. To achieve this, various malic acid accumulation modules were systematically evaluated and optimized. Additionally, glucose-6-phosphate isomerase, a key enzyme in the xylulose monophosphate (XuMP) pathway, was knocked out to release metabolic fluxes trapped in this cycle. The latter approach resulted in the accumulation of 2.79 g L^−1^ malic acid when using methanol as feedstock together with optimizing the nitrogen source [47].

Likewise, the mentioned advances in genomic-editing tools have led to the exploitation of *H. polymorpha*-based processes. For example, the production of various recombinant proteins such as Hepatitis E virus-like particles [48] or ferritin (FTH1) [49] from methanol or a glycerol/methanol mixture was achieved. Moreover, several commercially available hepatitis B vaccines and other biopharmaceuticals such as hirudin, insulin and IFNa-2a Reiferon® are produced using *H. polymorpha* [50].

The presented examples demonstrate the versatility of biotechnological applications using methylotrophic yeasts and demonstrate the potential to produce such and similar products using pure methanol as the substrate.

During the last years, there has also been an interest in methylotrophy and its application in white biotechnology as a potential silver bullet against climate change [51]. Various findings demonstrate that microorganisms play a key role in natures carbon cycle [52] and it is therefore speculated that they can support global climate change mitigation. Selected methylotrophic microbes have the capability to utilize methane as a carbon source. Such organisms help to reduce greenhouse gas concentration in the atmosphere [53]. In addition, liquid C_1_ substrates, sustainably produced from CO_2_, used for the production of bulk chemicals via fermentation can pinpoint the direction towards a cyclic bioeconomy to reduce mankind’s greenhouse gas emission footprint while providing economic benefits. Already in the early 2000s, the application of methylotrophic yeasts in the agricultural sector as biofertilizers and for the treatment of the methanol and formaldehyde containing wastewater was shown [54, 55].

Furthermore, the biotechnological production of high-energy fuels by economically feasible processes has emerged as an attractive alternative to the traditional production [56]. One promising approach exploits *P. pastoris* for the production of the platform chemical and potential biofuel isopentanol. Here, the authors heterologously expressed the keto-acid degradation pathway to convert 2-ketoisocaproate to isopentanol and reduced the production of the side-product ethanol via using the CRISPR/Cas9 system to delete PDC1. Consequently, 191 mg L^−1^ of isopentanol were produced, so far the highest reported titer in a non-conventional yeast [57].

Lately, *P. pastoris* was engineered towards CO_2_ consumption via the Calvin–Benson–Bassham cycle, the primary natural CO_2_-fixation pathway of photosynthetic organisms. By introduction of eight heterologous genes *P. pastoris* was converted into an autotroph capable to use CO_2_ as its single carbon source. To separate the foreign fixation machinery of CO_2_ from energy generation, the first steps of the XuMP pathway (AOX1, DAS1 and DAS2) were deleted. Following laboratory evolution, the engineered strain achieved a maximum growth rate of 0.018 h^−1^ [58]. Examples like these may form the basis for producing bulk- and fine-chemicals based on a sustainable CCU biotechnology and might support mitigation of atmospheric CO_2_ in the future.

## C_1_-metabolism in methylotrophic yeasts

**Fig. 2:**
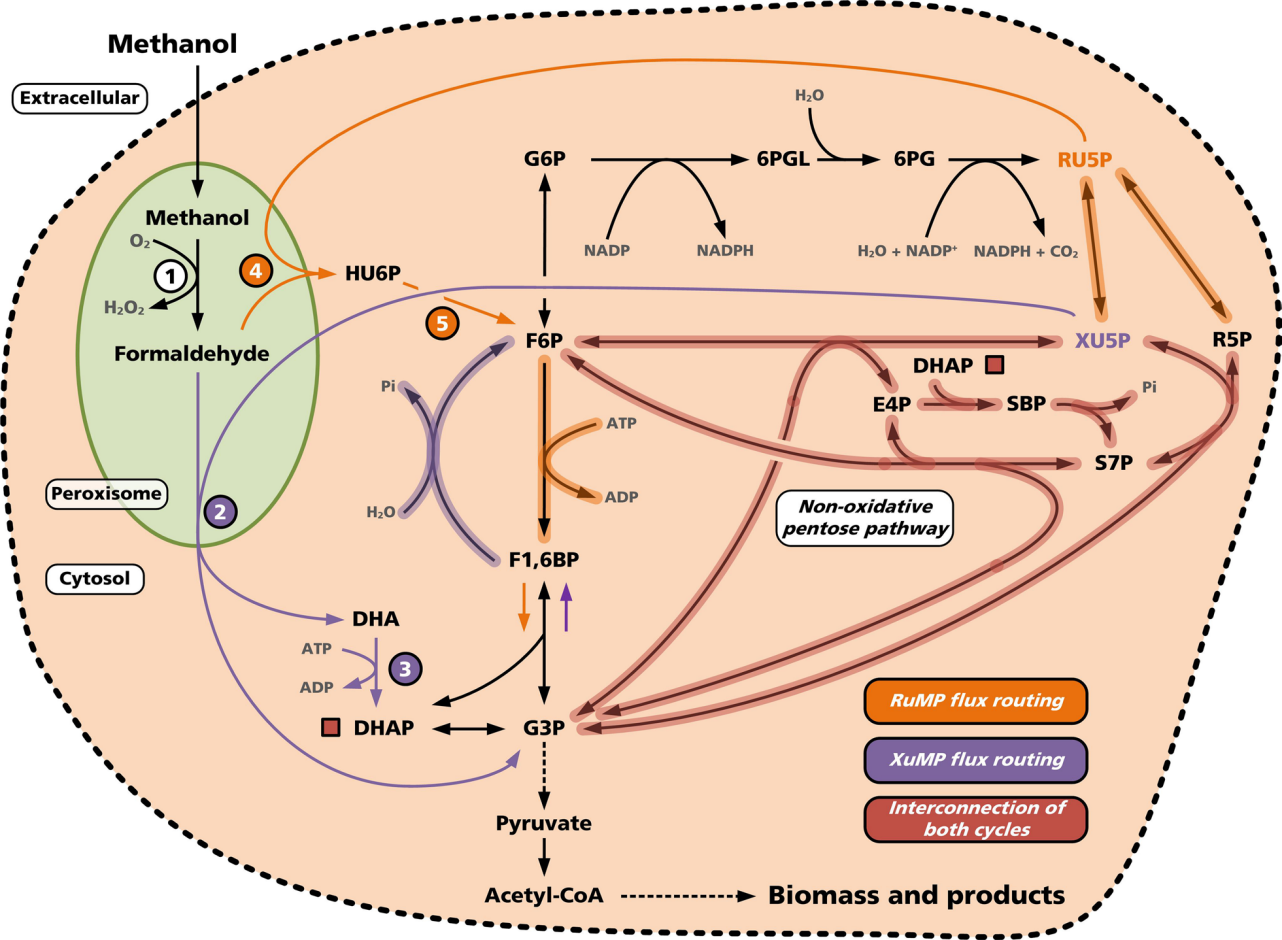
Traditional metabolic architecture comparison of the yeast-specific XuMP pathway and the bacteria-specific RuMP cycle based on the assumption that reactions take partly place in the cytosol. The flux routing of both pathways is highlighted as shaded arrows (purple, XuMP; orange, RuMP). It has to be underlined that both pathways share all reactions of the non-oxidative pentose phosphate pathway. The figure highlights in this context the distinct routes and the interconnection. Therefore, both pathways rely closely on the regeneration of a pentose, xylulose 5-phosphate or ribulose 5-phosphate, respectively, which are condensed with formaldehyde for C_1_-assimilation. Key enzymes of methanol oxidation, XuMP and RuMP are shown in white, purple or orange circles, respectively. AOX: alcohol oxidase (1); DAS: dihydroxyacetone synthase (2); DAK: dihydroxyacetone kinase (3); HPS: hexulose-6-phosphate synthase (4); PHI: hexulose-6-phosphate isomerase (5). Metabolite abbreviations are: G6P: glucose 6-phosphate; 6PGL: 6-phosphogluconolactone; RU5P: ribulose 5-phosphate; HU6P: hexulose 6-phosphate; R5P: ribose 5-phosphate; XU5P: xylulose 5-phosphate; S7P: sedoheptulose 7-phosphate; SBP: sedoheptulose 1,7-bisphosphate; E4P: erythrose 4-phosphate; F6P: fructose 6-phosphate; F1,6BP: fructose 1,6-bisphosphate; G3P: glyceraldehyde 3-phosphate; DHA: dihydroxyacetone; DHAP: dihydroxyacetonephosphate; O_2_: molecular oxygen; CO_2_: molecular carbon dioxide; H_2_O_2_: molecular hydrogen peroxide; NAD(P)^+^: oxidized nicotinamide adenine dinucleotide (phosphate); NAD(P)H: reduced nicotinamide adenine dinucleotide (phosphate); ADP: adenosine diphosphate; ATP: adenosine triphosphate; Pi: inorganic phosphate

The C_1_-metabolism of methylotrophic yeasts compared to bacteria differs primarily in the recruited enzymes, energy generation and carbon assimilation pathways. In general, methanol is oxidized to formaldehyde, which then can be diverted to either assimilatory (product biomass) or dissimilatory (product CO_2_) pathways (Fig. 2). Formaldehyde plays a pivotal role in the metabolism of methylotrophic organisms for various reasons. Precisely, (I) formaldehyde is generated mainly from methanol in the cell; (II) this molecule depicts the branch point between C_1_-assimilation and dissimilation in methylotrophic yeasts [59, 60]; and (III) it is an extremely toxic compound that non-specifically interacts with proteins and nucleic acids in all biological cells [61, 62]. Therefore, all methylotrophic organisms must mitigate formaldehyde toxicity during growth on methanol by maintaining low intracellular formaldehyde concentrations that can be quenched either by the assimilation or dissimilation pathway [63]. For assimilation, methylotrophic yeasts commonly use the XuMP pathway. Here, the first step of methanol oxidation is conducted via an unspecific peroxisomal O_2_-dependent alcohol oxidase (AOX) [64]. In detail, AOX has a molecular mass of 600 kDa and the crystalline structure consists of eight identical subunits of 74 kDa, each containing a flavin adenine dinucleotide molecule (FAD) as the prosthetic group [65]. The oxidation of methanol leads to the formation of formaldehyde and hydrogen peroxide. Both pose a toxic challenge for cells, addressed by eukaryotic compartmentalization in the peroxisomes. This compartmentation is thought as a result of evolution to separate toxic formaldehyde formation and detoxification processes of the cumulating hydrogen peroxide from the cytosol [13]. In detail, the peroxisomal enzyme catalase (CAT) together with the small protein peroxiredoxin (Pmp20) decomposes the hydrogen peroxide to water and oxygen [66]. However, AOXs require aerobic conditions and exhibit a higher methanol oxidation efficiency (Δ_r_*G* = − 127.5 kJ mol^−1^) compared to PQQ-dependent MDHs (Δ_r_*G* = 59.1 kJ mol^−1^) or NAD^+^-dependent MDHs (Δ_r_*G* = − 0.4 kJ mol^−1^), calculated under physiological conditions (37 °C, 1 bar, pH 7.6, 0.1 M ionic strength and 987.5 mM MeOH) [67].

A detailed understanding of formaldehyde assimilation and dissimilation pathways in methylotrophic microbes is crucial for the establishment of synthetic methylotrophic modules in yeasts.

### Carbon assimilation during methylotrophic growth

In methylotrophic yeasts, formaldehyde is assimilated using the dihydroxyacetone (DHA) pathway, also known as the XuMP pathway [68]. A comparison to the ribulose monophosphate (RuMP) pathway, found predominantly in bacteria, is depicted in Fig. 2. In the first step (peroxisomal), formaldehyde is condensed with xylulose 5-phosphate (Xu5P) by the peroxisomal key enzyme DAS (dihydroxyacetone synthase). Two C_3_ compounds are formed in this reaction, namely dihydroxyacetone (DHA) and glyceraldehyde 3-phosphate (G3P) to fuel gluconeogenic reactions [69]. Subsequently, DHA and G3P are released from the peroxisomes into the cytosol [70]. Recently, it was revealed that the cytosolic localization might not occur in *P. pastoris*. It was shown by omics-level investigations of the metabolism that this yeast orchestrates all assimilation steps within the peroxisome [69]. Next, the cytosolic DHA is phosphorylated to dihydroxyacetone phosphate (DHAP) by a dihydroxyacetone kinase (DAK), the second key enzyme of the XuMP pathway [71]. The latter reaction cascade connects the C_1_-metabolism with the common central carbon metabolism on the level of glycolysis by formation of fructose 1,6-bisphosphate (F1,6BP) from DHAP and G3P. Subsequently, fructose 6-phosphate (F6P) is formed by dephosphorylating F1,6BP, connecting the former C_1_-assimilation to gluconeogenesis and the pentose phosphate pathway [69]. In particular, the F6P pool is partly harnessed for Xu5P regeneration by recruiting the non-oxidative pentose phosphate pathway branch and the associated pentose interconversion reactions. It has to be highlighted that the recruiting of a transketolase yields erythrose 4-phosphate and Xu5P from F6P and G3P. Strikingly, a distinct feature of methylotrophic pentose rearrangements is the subsequent conversion of erythrose 4-phosphate (C_4_) into sedoheptulose 1,7-bisphosphate (C_7_) using DHAP (C_3_) by application of an aldolase. Finally, sedoheptulose 1,7-bisphosphate is dephosphorylated to sedoheptulose 7-phosphate, which is in turn converted by a transketolase and G3P into two Xu5P units [69].

In consequence, the molecule sedoheptulose 1,7-bisphosphate and sedoheptulose 7-phosphate are central intermediates for Xu5P regeneration in comparison to the traditional non-oxidative pentose phosphate branch. The fact that Xu5P is directly regenerated within the peroxisomes makes its import unnecessary and thus improving the efficiency of formaldehyde assimilation. Previously, it was thought that the import of Xu5P into the peroxisome is strictly necessary [69, 72].

The pentose regeneration is strictly required to maintain efficient methanol assimilation [73] by regeneration of the formaldehyde-acceptor pentose unit (Xu5P for XuMP and Ru5P for RuMP, respectively) to keep the XuMP, and RuMP pathway running [70]. However, the remaining carbon fraction of the G3P pool is finally utilized for biosynthesis of cell constituents via pyruvate and acetyl-CoA (Fig. 2).

During growth on methanol, the peroxisomes of methylotrophic yeasts massively proliferate accompanied by high AOX and DAS expression [74]. Juxtaposed, when grown on other carbon sources, the enzymatic activities of AOX and DAS are not detectable, indicating that both genes of AOX and DAS are induced in presence of methanol [21, 75]. While the specific mechanism of this overexpression under methanol abundant conditions is not elucidated to date, deletions in several methanol-inducible promoter sequences lead to the identification of cis-acting elements thought to play a role in gene regulation. For AOX regulation, significant differences were found among the different methylotrophic yeast strains, which seemed to be mainly due to the regulatory mechanism of the host rather than the promoter regions [76].

### Carbon dissimilation during methylotrophic growth

In order to keep the intracellular formaldehyde levels low, not only assimilation into biomass but also the dissimilation towards CO_2_ takes place. The dissimilation is closely related to redox power generation. Particularly, the dissimilation functions as a valve to cope with toxic intracellular formaldehyde concentrations while covering NAD(P)H demand. The most frequent pathway for formaldehyde detoxification is the cytosolic thiol-dependent pathway, which employs reactive thiols as the initial formaldehyde acceptor [77]. This pathway generates redox power (i.e., NADH) and is used by methylotrophic bacteria and other non-methylotrophic organisms [78]. The produced NADH is used in cellular respiration to sustain the generation of ATP in presence of formaldehyde and supports the energy demand of the cell. Specifically, formaldehyde spontaneously reacts in the peroxisomes with glutathione (GSH) and generates S-hydroxymethylglutathione (S-HMG) [79], which is oxidized to CO_2_ in a subsequent cytosolic GSH-dependent oxidation cascade. In detail, the S-HMG is released from the peroxisomes into the cytosol and is oxidized to S-formylglutathione (S-FG) via a NAD^+^-linked and GSH-dependent formaldehyde dehydrogenase (FLD), which is shown to be essential for growth of *C. boidinii* on methanol [78]. Subsequently, S-FG is hydrolyzed via S-formylglutathione hydrolase (FGH) to formate. In the dissimilatory branch, a formate dehydrogenase (FDH) oxidizes the generated formate to CO_2_ accompanied by NADH formation. In turn, the role of FDH is not only the formaldehyde detoxification but also retaining the redox-state and the regulation of the glutathione level in cells. However, it was demonstrated, that FDH is not essential for growth on methanol in *C. boidinii*. Nonetheless, as the complete genome is not yet sequenced, the existence of other FDHs cannot be excluded that supported growth during the study [78]. The latter is in contrast to the fact that FDH proteins from methylotrophic yeasts are very stable enzymes and represent about 10 to 18% of the total cellular proteins [80].

It is still not completely understood how the efficient and dynamic distribution of formaldehyde between assimilatory and dissimilatory metabolism without toxic accumulation is conducted. However, it can be stated that compartmentalization of peroxisomal methylotrophy is highly beneficial for methylotrophic yeasts. Juxtaposed, for bacteria the formaldehyde distribution is a challenge in regard of balancing metabolic fluxes into the dissimilatory and assimilatory branch, to avoid formaldehyde accumulation.

### The branch point between assimilation and dissimilation

While in the XuMP pathway formaldehyde represents the central intermediate, some studies concluded that in methylotrophic bacteria formate is the branch point between assimilatory and dissimilatory pathways [81]. Here, the significance of the direct condensation route for methylene H_4_F synthesis in *M. extorquens AM1* was assessed. It was indicated, that during laboratory growth conditions, methylene H_4_F is originally formed from formaldehyde via the H_4_MPT and H_4_F interconversion pathway. The latter suggests that indeed formate and not formaldehyde represents the primary metabolic branch point between assimilation and dissimilation of C_1_ units in this microbe [81]. In turn, this additionally indicates that the spontaneous condensation of formaldehyde with H_4_F does not occur in vivo, which was confirmed recently [82, 83].

Other studies consider both formaldehyde and formate as key intermediates of the bacterial methylotrophic metabolism [84]. Specifically, formaldehyde represents the initial branch point via the split of linear oxidation towards CO_2_ or the recruitment by central carbon metabolism using the RuMP pathway. Here, formaldehyde is primarily diverted to biomass formation [85]. Moreover, it has been detected that a partial serine cycle exists in gammaproteobacterial methanotrophs, which might contribute to linear formaldehyde oxidation and carbon conversion to acetyl-CoA [86]. An accumulation of the toxic intermediate formate leads to a stress response and hampers growth in microorganisms [87]. This formate accumulation during methylotrophic growth led to the postulation that formate could be utilized by oxidization to CO_2_ for NADH generation as well as incorporation into the serine cycle. Moreover, it is also described that formaldehyde oxidation to formate in bacteria has a much greater capacity than methanol oxidation to avoid toxic formaldehyde accumulation [88].

### Design aspects of synthetic methylotrophy

**Fig. 3:**
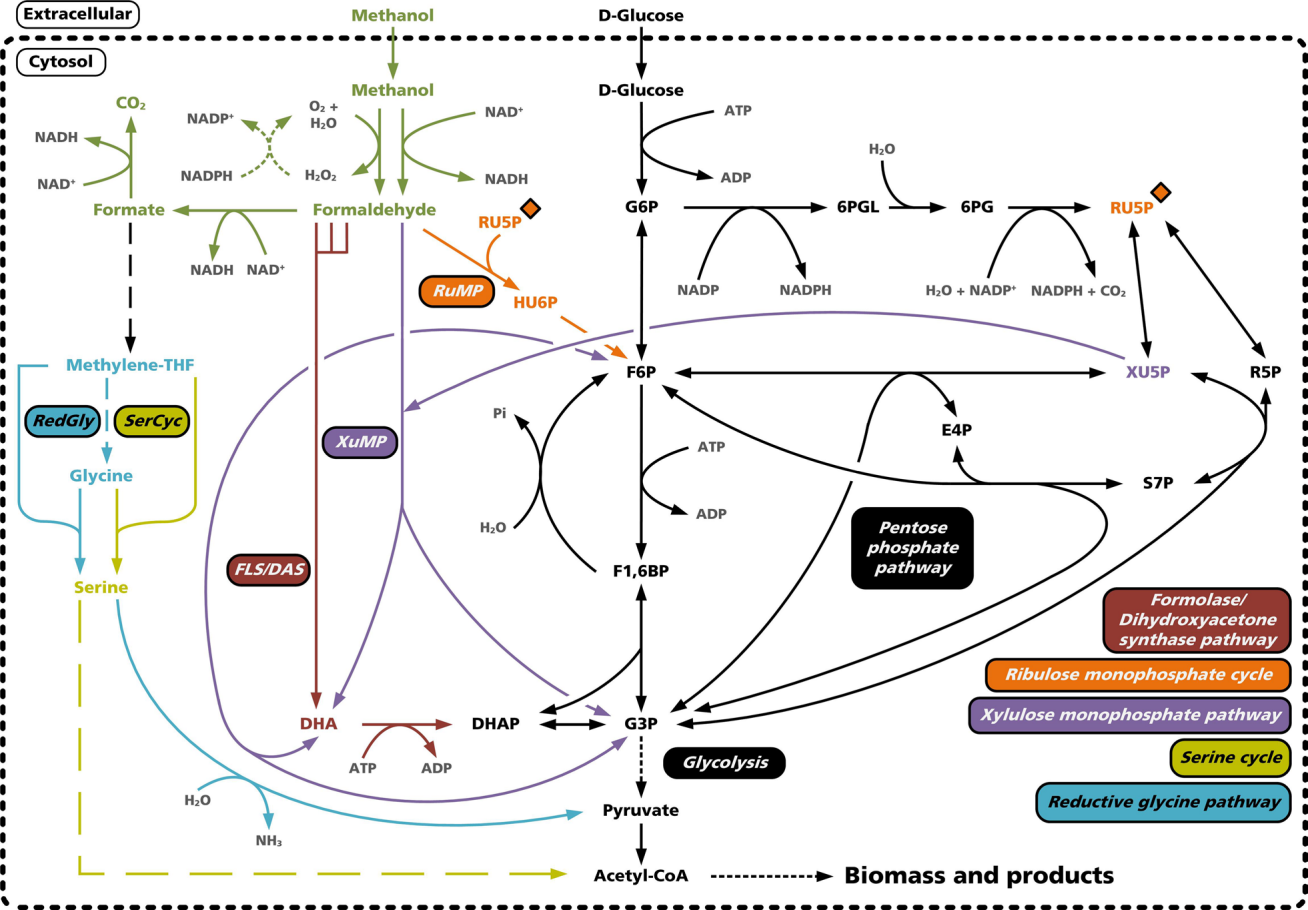
Metabolic access points of synthetic and native methylotrophic pathways into glycolytic yeast metabolism. Shown is the metabolic overlap of named methylotrophic and glycolytic pathway architectures to funnel C_1_ substrates like methanol into central carbon metabolism as main target of synthetic methylotrophy. Depicted methylotrophic pathways are limited to the main important variants. Orange diamond marks the connection of RuMP cycle with formaldehyde assimilation via the regenerated pentose. Metabolite abbreviations are: G6P: glucose 6-phosphate; 6PGL: 6-phosphogluconolactone; RU5P: ribulose 5-phosphate; HU6P: hexulose 6-phosphate; R5P: ribose 5-phosphate; XU5P: xylulose 5-phosphate; S7P: sedoheptulose 7-phosphate; E4P: erythrose 4-phosphate; F6P: fructose 6-phosphate; F1,6BP: fructose 1,6-bisphosphate; G3P: glyceraldehyde 3-phosphate; DHA: dihydroxyacetone; DHAP: dihydroxyacetonephosphate; O_2_: molecular oxygen; CO_2_: molecular carbon dioxide; H_2_O: molecular water; H_2_O_2_: molecular hydrogen peroxide; NAD(P)^+^: oxidized nicotinamide adenine dinucleotide (phosphate); NAD(P)H: reduced nicotinamide adenine dinucleotide (phosphate); ADP: adenosine diphosphate; ATP: adenosine triphosphate; NH_3_: molecular ammonia; P_i_: inorganic phosphate

When designing synthetic methylotrophic hosts, not only pathway kinetics and accumulation of toxic intermediates but also the stoichiometry of carbon and energy conservation have to be considered. While the serine cycle achieves the highest yield of the metabolic precursor pyruvate, it also has the highest metabolic costs in terms of ATP usage (Table 1). The RuMP cycle and XuMP pathway yield slightly less pyruvate but, in contrast, form ATP, thus providing energy supply [89]. Regarding ATP generation, the XuMP pathway is the most promising option. Nevertheless, the corresponding AOX requires the presence of oxygen and is located in the peroxisomes, which might be a drawback depending on the used host and desired production route. Besides the mentioned natural methanol assimilation pathways, also synthetic alternatives exist (Fig. 3). The reductive glycine pathway or the artificial FLS pathway, among others, depict further metabolic access points into glycolytic yeast metabolism to establish synthetic methylotrophy.

**Table 1:** Overview of formaldehyde assimilation pathways and their characteristics

Pathway	Characteristics	Pyruvate and ATP yield	Refs.
RuMP	Cyclic assimilation pathway found in bacteria, formaldehyde enters the RuMP cycle through condensation with Ru5P	0.33 mol_pyruvate_ mol_methanol_^−1^ 0.33 mol_ATP_ mol_methanol_^−1^	[90]
Serine cycle	Cyclic assimilation pathway found in bacteria, formaldehyde enters the pathway through methylene H4F	0.5 mol_pyruvate_ mol_methanol_^−1^ -1 mol_ATP_ mol_methanol_^−1^	[67]
XuMP	Cyclic assimilation pathway found in yeasts, compartmentalization in the peroxisomes, formaldehyde enters the pathway through condensation with Xu5P	0.33 mol_pyruvate_ mol_methanol_^−1^ 0.66 mol_ATP_ mol_methanol_^−1^	[91]

All these considerations should be taken into account when designing synthetic methylotrophic engineering projects, but they also demonstrate that there is still a need for research in the field of methylotrophy in general.

## Synthetic methylotrophic yeasts: chances and challenges

But why is the installation of methylotrophy in non-methylotrophic microorganisms a current trend? When comparing glucose and methanol, the latter is a promising non-food C_1_ feedstock which supports increased biomass and product yields. In addition, the oxidative combustion of methanol provides more energy (Δ*G*°′ = − 4276.6 kJ mol^−1^) compared to glucose oxidation (Δ*G*°′ = 2870 kJ mol^−1^) [92]. Therefore, the implementation of synthetic methylotrophy into conventional and established microbial host organisms depicts an attractive alternative to switch the feedstock basis [93].

Although notable progress has been made regarding the availability of genetic tools for native methylotrophic organisms, many of them are still not adequate characterized or their intrinsic capabilities to efficiently produce high value-added chemicals are limited. Besides that, a fundamental knowledge about the physiology, the genome and the metabolism is crucial for successful metabolic engineering of such microbes. Many of these aspects lack a robust basis when considering methylotrophic yeasts for engineering efficient microbial cell factories.

In consequence, industrial glycolytic yeasts or bacteria depict promising host organisms to exploit synthetic methylotrophy for efficient production of value-added products from C_1_ substrates. Due to the long tradition of investigating such microbes, the knowledge base and available engineering tools are fundamentally broad and established to realize synthetic methylotrophy [11]. Furthermore, platform organisms like *S. cerevisiae* or the oleaginous yeast *Y. lipolytica* have the ability or were engineered to produce industrially relevant products such as bulk chemicals (e.g., monoalcohols, diols, organic acids, biopolymers) or biofuels and precursors of biofuel molecules (e.g., alcohols, alkanes, carboxylic acids, fatty acids) with increased yield and titer (Table 2). In regard of the various engineered producer strains, it is logical to switch food-related sugar substrates against methanol. This approach can enable a more sustainable, and even CO_2_-based, production of important chemical products via fermentation.

**Table 2:** State-of-the-art yeast producer strains for the production of important biofuels and bioproducts from conventional feedstock as potential host organisms for implementation of synthetic methylotrophy

Host organism	Product	Procedure	Substrate	Titer	Refs.
*S. cerevisiae*	Prenyl alcohols	Overexpression of the gene encoding hydroxymethylglutaryl (HMG)-CoA reductase. Production of (*e,e*)-farnesol (FOH), (*e*)-nerolidol (NOH), and (*e,e,e*)-geranylgeraniol (GGOH))	5% galactose, addition of glucose after 125 h (5% final concentration)	145.7 mg L^−1^ (FOH), 98.8 mg L^−1^ (NOH), and 2.46 mg L^−1^ GGOH	[94]
*S. cerevisiae*	Bisabolene	Overexpression of truncated HMG-CoA reductase (tHMGR), the FPP synthase (Erg20), and the global transcription regulator of the sterol pathway upc2-1, downregulation of the squalene synthase (Erg9)	1.8% galactose/0.2% glucose	> 900 mg L^−1^	[95]
*S. cerevisiae*	Medium chain fatty acids C6-C10	Endogenous fatty acid synthase (FAS) and an orthogonal bacterial type I FAS were engineered for MCFA production in the yeast *S. cerevisiae*. Directed evolution of the membrane transporter Tpo1 and adaptive laboratory evolution of the strain	Various glucose concentrations	> 1 g L^−1^	[96]
*S. cerevisiae*	*n*-Butanol	*S. cerevisiae* was engineered with a n-butanol biosynthetic pathway with isozymes from a number of different organisms (*S. cerevisiae, E. coli, Clostridium beijerinckii*, and *Ralstonia eutropha*)	2% galactose	2.5 mg L^−1^	[97]
*S. cerevisiae and P. stipitis*	Ethanol	Recursive protoplast fusion of two yeast strains	200 g L^−1^ glucose–xylose mixture (3:1 ratio)	74.65 g L^−1^	[98]
*S. cerevisiae*	Vitamin E tocotrienols	Expression of HPPD, HGGT, MPBQMT, TC and γ-TMT from photosynthetic organisms and design of a cold-shock-triggered temperature control system used in a two-stage fermentation	3% glucose	320 mg L^−1^	[99]
*S. cerevisiae*	Hemoglobin	Deletion HMX1, VPS10, PEP4 and ROX1 and overexpression of HEM3 and AHSP genes	2% glucose	18% (of total cell protein)	[100]
*Y. lipolytica*	Citric acid	Elongation of the production phase of the bioprocess with growth-decoupled citric acid production	1.5% glucose	~ 100 g L^−1^	[101]
*Y. lipolytica*	Omega-3 eicosapentaenoic acid	Overexpression of the ∆9/∆8 pathway (41 copies of 19 different genes) and optimization of lipid metabolism	2% glucose	25% of yeast biomass	[102]
*Y. lipolytica*	Fatty acid ethyl esters	Expression of pyruvate decarboxylase (*pdc*) and alcohol dehydrogenase II (*adhB*) from *Zymomonas mobilis* and introduction of heterologous wax ester synthases *ws2* and *maqu_0168* from disruption of competitive pathways to increase fatty acyl-CoA pool	2% dextrose	8.2 mg L^−1^	[103]
*Y. lipolytica*	1-Decanol	Overexpression of FAR from *Arabidopsis thaliana* and FAT from *C. palustris*. Deletion of the major peroxisome assembly factor Pex10	5% glucose	> 500 mg L^−1^	[104]
*Y. lipolytica*	FAEE	Expression of WS gene from *Marinobacter sp.* and deletion of *PEX10* gene	2–6% glucose, 2–10% ethanol	1.18 g L^−1^	[105]
*Y. lipolytica*	FFAs	Overexpression of hybrid hFAS-EcTesA	10% glucose	9.67 g L^−1^	[106]
*Y. lipolytica*	β-carotene	Overexpression of β-carotene pathway and promoter screening	6 g h^−1^ glucose	6.5 g L^−1^	[107]
*Y. lipolytica*	Docosahexaenoic acid	Expression of artificial *pfa* BGC from *Aetherobacter fasciculatus*	2.5% glucose	350 mg L^−1^	[108]
*Y. lipolytica*	Cyclo-propane fatty acids	Overexpression of the *E. coli* cyclopropane fatty acid synthase gene under a hybrid promoter (hp8d) and *Y. lipolytica* LRO1 gene	7% glucose	3.06 g L^−1^	[109]

Especially with regard to major concerns about global climate change and increasingly difficult access to fossil fuels, synthetic methylotrophy has taken up the challenge to produce advanced biofuels and bioproducts. To this extent, expanding the substrate scope of the organism by the design and implementation of non-native carbon assimilation pathways is promising. Such an approach introduces the required enzymes and pathway modules into established industrial hosts. Subsequently, understanding and fine-tuning of redox balances, energy metabolism, carbon-fluxes as well as the transcriptional and translational regulation is mandatory for successful engineering projects to achieve beneficial efficiencies [110].

### Synthetic methylotrophic bacteria—a blueprint for yeasts?

Recently, tremendous progress in implementing synthetic methylotrophic pathway modules on genetic level into different bacteria, such as *E. coli* and *C. glutamicum* was made [111, 112]. Therefore, it is likely to use these examples as blueprints for engineering synthetic methylotrophic yeasts. But besides the published successes, challenges remain still to date.

Even though incorporation of labeled carbon from ^13^C-methanol into biomass building blocks was proven in *E. coli* as well as *C. glutamicum*, growth on methanol as the sole carbon source still required yeast extract or additional sugars as energy or carbon source [67, 113]. To overcome this limitation, a recombinant autotrophic *E. coli* strain was presented, which harnesses formate as its sole energy source for generating redox power to build up biomass completely from CO_2_ by heterologous implementation of the Calvin cycle [114]. In detail, overexpression of FDH, Rubisco and phosphoribulokinase (PRK) enabled autotrophic growth. Initial growth experiments failed while adaptive laboratory evolution (ALE) was the key to convert the engineered strain into a fully autotrophic organism. Isotopic labeling of biomass constituents using ^13^CO_2_ or ^13^C-formate, either solely or in combination proved the autotrophic growth mode. However, from an academic viewpoint these results are interesting towards true synthetic autotrophy, but far away from a real application. Another advanced example was achieved through the de novo design of a synthetic pathway in *E. coli* to produce acetyl-CoA from formaldehyde. It was proven that this synthetic acetyl-CoA pathway (SACA) is the shortest, ATP-independent, carbon-conserving and oxygen-insensitive pathway for acetyl-CoA biosynthesis from a C_1_ feedstock. The latter opens enormous possibilities for producing acetyl-CoA-derived chemicals from renewable one-carbon resources [115]. Furthermore, *E. coli* was engineered towards growth on one-carbon compounds using the reductive glycine pathway. Integration of the synthetic pathway coupled to laboratory evolution enabled growth on formate and CO_2_ with a doubling time of ~ 8 h and growth yield of ~ 50 mg cell dry weight (CDW) g formate^−1^. Furthermore, growth on methanol and CO_2_ was achieved by expressing a methanol dehydrogenase, resulting in a further increased doubling time (54 ± 5.5 h), due to the slow methanol oxidation rate [116]. This study is the first example that demonstrates true synthetic methylotrophy in a non-methylotrophic host strain.

The reviewed advances in the bacterial phyla are a promising blueprint for adaptation into conventional yeasts to establish synthetic methylotrophy in eukaryotic hosts, opening the door for additional applications, products and processes. However, it has to be respected that the eukaryotic compartmentalization is also challenging when considering prokaryotic engineering strategies. Furthermore, several factors can affect the expression yield of recombinant enzymes in yeasts. When expressing bacterial genes in yeasts often codon optimization is required to achieve faster translation rates and high accuracy [117].

### The road towards synthetic methylotrophy in baker’s yeasts

Beside bacterial hosts, well established model organisms like *S. cerevisiae* or other industrially relevant yeast, which were shown to produce various products with high titers, exhibit a vast potential as hosts for synthetic methylotrophy [92].

Recently, the installation of synthetic methylotrophic modules in *S. cerevisiae* was carried out [118]. In detail, three different metabolic pathways were applied. The native methylotrophic yeast XuMP pathway was implemented and the expression of the associated enzymes was targeted to the peroxisomes.

The latter strategy resulted in a subtle growth increase on agar plates containing YNB and 1% methanol compared to the empty vector control. In subsequent steps, engineering of a ‘hybrid’ XuMP pathway including a NAD^+^ dependent MDH, or a bacterial RuMP pathway was conducted. Subsequently, methanol toxicity assays and ^13^C-methanol labeling demonstrated basic functionality of the bacterial RuMP pathway. In addition, this variant seemed to be the most promising synthetic pathway, indicated by the growth profile and the increased ^13^C-CO_2_ production levels.

Surprisingly, at higher substrate concentrations striking methanol assimilation was observed in the wild-type strain. This C_1_-assimilation was proven by ^13^C-ethanol production from ^13^C-methanol. The latter suggests that *S. cerevisiae* possesses native capacities for methanol oxidation and assimilation. Such findings offer new opportunities to advance microbial strain development of both, native and synthetic, one-carbon assimilation pathways in this organism [118]. In particular, identification of unknown associated enzymes, pathways or regulative mechanisms can help to understand, and engineer the native methanol assimilation.

Following the modular approach, another study demonstrated implementation of synthetic methylotrophy in *S. cerevisiae* and tested in vivo methanol assimilation. The strain engineering relied on genomic integration of AOX, catalase (CAT), dihydroxyacetone synthase (DAS) and dihydroxyacetone kinase (DAK) derived from *P. pastoris.* In subsequent growth experiments, the engineered strain consumed 1.04 g L^−1^ methanol applying shake-flask conditions with synthetic medium. The yeast produced 0.26 g L^−1^ pyruvate and exhibited a 3.13% improvement of biomass formation in methanol minimal medium compared to the wild-type strain. Consistent with previous findings, the supplementation of yeast extract improved methanol consumption even further to 2.35 g L^−1^ and cell growth by 11.7%, respectively [119]. This growth-enhancing effect of yeast extract supplementation in synthetic methylotrophy is commonly found indicating that complex media components can support synthetic methanol metabolism. Especially, biosynthesis of amino acids or vitamins and cofactors can play a key role for the observed growth dependencies [69, 120].

To further enhance synthetic methylotrophic capabilities of *S. cerevisiae*, the model strains S288C and CEN.PK were investigated in terms of growth and transcriptomic responses to methanol. The strain CEN.PK showed improved growth and the upregulation of genes linked to mitochondrial and peroxisomal metabolism, alcohol and formate oxidation and the *mig3* gene. The rational overexpression of the *mig3* gene improved furthermore the methanol-dependent growth in CEN.PK, generating a superior strain for future synthetic methylotrophic applications [121].

Lately, it was verified that *S. cerevisiae* has a native capacity for methylotrophy. Native methanol assimilation was confirmed through ^13^C-tracer analysis and further improved by applying ALE. It was demonstrated that global rearrangements in central carbon metabolism and a truncation of the transcriptional regulator Ygr067cp improved growth on methanol. Nevertheless, also in this study the requirement for yeast extract in liquid methanol medium still remains a challenge [122]. Recent findings have shown that the connection between pentose phosphate pathway is essential for synthetic methylotrophy on the one hand for pentose regeneration of RuMP and XuMP pathway and on the other hand for the synthesis of complex biomass precursors or vitamins and cofactors [123].

### Is pentose regeneration the key to synthetic methylotrophy?

Beside the oxidative pentose phosphate pathway branch, yielding mainly NADPH for assimilatory reactions, the non-oxidative pentose phosphate pathway branch has also a central role in methanol assimilation during methylotrophy [69]. In particular, the pentose rearrangement reactions are of importance to regenerate the metabolites Xu5P and Ru5P [124]. Specifically, both molecules are used as acceptors for formaldehyde assimilation in XuMP and RuMP cycle, respectively [59]. Therefore, the depletion of these metabolites has vast influence on the pathway efficiencies. In consequence, constant replenishment of the pentose pools has to be ensured by appropriate metabolic flux distribution to drive the individual assimilation cycles of XuMP and RuMP. As indicated in Figs. 2 and 3, the pentose rearrangement reactions are tightly interconnected and enable the flexible adaptation of the metabolic fluxes to replenish the Xu5P as well as Ru5P pool. However, it is stated that the replenishment of the pentoses originates from the fructose 6-phosphate pool instead of the oxidative pentose phosphate branch relying on glucose 6-phosphate and prior gluconeogenesis [69].

With regard to the latter aspects, it is remarkable that in *P. pastoris* (and presumably other methylotrophic yeasts) the Xu5P regeneration reactions of the XuMP cycle are located in the peroxisomes. This was recently shown by analyzing the systems-level organization of the *P. pastoris* metabolism [69].

In detail, the pentose phosphate pathway gene–protein pairs of the enzymes transaldolase (Tal1-2) and ribose-5-phosphate isomerase (Rki1-2) were upregulated. Furthermore, it was shown that the Fructose-1,6-bisphosphate aldolase 2 (Fba2p) and the Transaldolase 2 (Tal2p) from *P. pastoris* are methanol-inducible and possess a peroxisomal targeting signal (PTS1). It is speculated that the non-oxidative pentose phosphate pathway involving Tal2p functions in a complementary manner in the cytosol [125]. In contrast, the isoform of ribulose-5-phosphate 3-epimerase (Rpe1-2) was not found to be upregulated [69]. All protein sequences of the related enzymes provide a PTS1 peroxisomal targeting signal, indicating the potential localization in the peroxisomes. Juxtaposed, the cytosolic and mitochondrial isoforms (Fba1-1, Tal1-1, Rki1-1, and Rpe1-1) do not show a peroxisomal targeting sequence and are not upregulated. In consequence, it can be assumed that the peroxisomal, in comparison to the cytosolic/mitochondrial, non-oxidative pentose phosphate branch plays a major role during methylotrophy in yeasts and uses another mode of action in comparison to glycolytic metabolic traits.

In particular, it is interesting that the interconversion of Xu5P and Ru5P is of minor importance due to the same differential Rpe1-2 expression profiles. This fits the assumption that replenishment of Ru5P within the XuMP cycle via these reactions of the non-oxidative pentose phosphate branch is in general not necessary due to the central role of Xu5P [69]. Taken together, it can be speculated that flux routing of the non-oxidative pentose phosphate branch, as indicated in Fig. 2, differs significantly between RuMP and XuMP.

However, another remarkable finding is that a second peroxisomal metabolic module relying on non-oxidative pentose phosphate pathway exists in *P. pastoris*, replenishing Xu5P by hydrolysis of sedoheptulose 1,7-bisphosphate to sedoheptulose 7-phosphate, which finally fuels the pentose pool [69]. The latter was proven by presence of sedoheptulose 1,7-bisphosphate in methanol-grown *P. pastoris* cells in contrast to the lack of this molecule in glucose-grown cells. Taken together, many aspects of the methylotrophic yeast metabolism were elucidated by studying *P. pastoris*, but further investigations are needed on fluxome level with solely methanol-grown cells to fully analyze and describe the flux routing of the pentose phosphate interconversions to fuel Xu5P replenishment. Such investigations, and in consequence the understanding of the exact flux distribution, can yield a blueprint for fine-tuning synthetic methylotrophic metabolism when installing the XuMP (or also RuMP) cycle.

In addition to pentose regeneration, the importance of the non-oxidative pentose phosphate branch is even deeper intertwined with the metabolism. It was shown that methanol utilization in *P. pastoris* is associated with the overproduction of vitamins and cofactors [69]. These molecules are required for the recruited enzymes. Here, the synthesis of flavin adenine mononucleotide or riboflavin are examples, which require pentoses as precursor metabolites [69]. Such findings can explain why almost all synthetic methylotrophs require small amounts of complex media components for growth on sole methanol as the carbon source.

In this regard the specific role of yeast extract and associated compounds that stimulate cell growth during synthetic methylotrophy is not fully known to date. Yeast extract is a complex hydrolysate of yeast biomass, which provides carbon, sulfur, trace nutrients, vitamin B complex and other important growth factors [126]. To release the growth-dependence of yeast extract, a synthetic methylotrophic *E. coli* strain was optimized in the absence of yeast extract in a laboratory evolution approach [111]. Initial depletion of yeast extract led to reduced growth. Strikingly, after nine passages, an increased optical density was reached. Interestingly, this biomass formation outcompeted even the unevolved strain using yeast extract supplementation. To understand the underlying principle mechanism, genome sequencing of the evolved strains resolved associated mutations in genes encoding glutathione-dependent formaldehyde oxidation (*frmA*), NAD(H) homeostasis/biosynthesis (*nadR*), phosphopentomutase (*deoB*), and gluconate metabolism (*gntR*) [111]. The identified mutations in *deoB* induced a genetic loss of function. This is remarkable since the associated enzyme represents a branch point in the RuMP cycle. It catalyzes the transfer of a phosphate group between the C_1_ and C_5_ carbon atoms of ribose or deoxyribose, respectively [127]. Even though the yeast extract supplementation is not yet fully understood, these data indicate again influence on the pentose phosphate pathway level.

Due to the complex interconnection of the pentose regeneration and the synthesis of vitamins and cofactors (or even other unknown aspects) a combination of metabolic engineering and systems-level analysis can support successful installation of synthetic methylotrophy in yeast. This approach was impressively demonstrated by elucidation of methylotrophic traits in *P. pastoris* on systems-level scale.

### Engineering of a non-conventional yeast for synthetic methylotrophy

Non-conventional yeast species like *Y. lipolytica* offer potential advantages over *S. cerevisiae* in terms of general substrate scope, metabolic pathway requirements, and physiological stress responses. It has a higher solvent tolerance in general and was shown to easily tolerate 4% methanol as a co-substrate [110].

Similar to *S. cerevisiae* also for the ascomycetous yeast *Y. lipolytica* a native capacity for methylotrophy in form of a non-specific alcohol dehydrogenase was proposed. A recent approach suggests using crude glycerol, which is contaminated with methanol, as a feedstock for engineered *Y. lipolytica.* In order to develop microbes, which use methanol as a co-substrate, the formaldehyde dehydrogenase (FLD) gene was identified and deleted. This prevents methanol dissimilation to CO_2_ via formaldehyde and formate. The generated deletion strain oxidized methanol to formaldehyde without the expression of a heterologous methanol dehydrogenase. To complement the Δ*fld1* strain, either HPS or DHAS were expressed and these designs enabled restoring the formaldehyde tolerance upon FLD deletion [128].

Another approach combined metabolic engineering with ALE. By rationally constructing a chimeric assimilation pathway in *Y. lipolytica*, engineering enhanced precursor supply, and ALE, improved methanol assimilation up to 1.1 g L^−1^ after 72 h was achieved. Here, a chimeric pathway, consisting of BsMDH, BmHPS, and BmPHI (RuMP pathway) and PpDAS1 and PpDAK2 (XuMP pathway), facilitated the most efficient methanol assimilation in *Y. lipolytica*. Furthermore, fine-tuning of methanol assimilation and enhancing formate dehydrogenation and serine pathways were exploited. In addition, upregulation of ribulose monophosphate/xylulose monophosphate (RuMP/XuMP) regeneration genes and subjecting the resulting strain to ALE were key towards improved methanol assimilation [110].

**Fig. 4:**
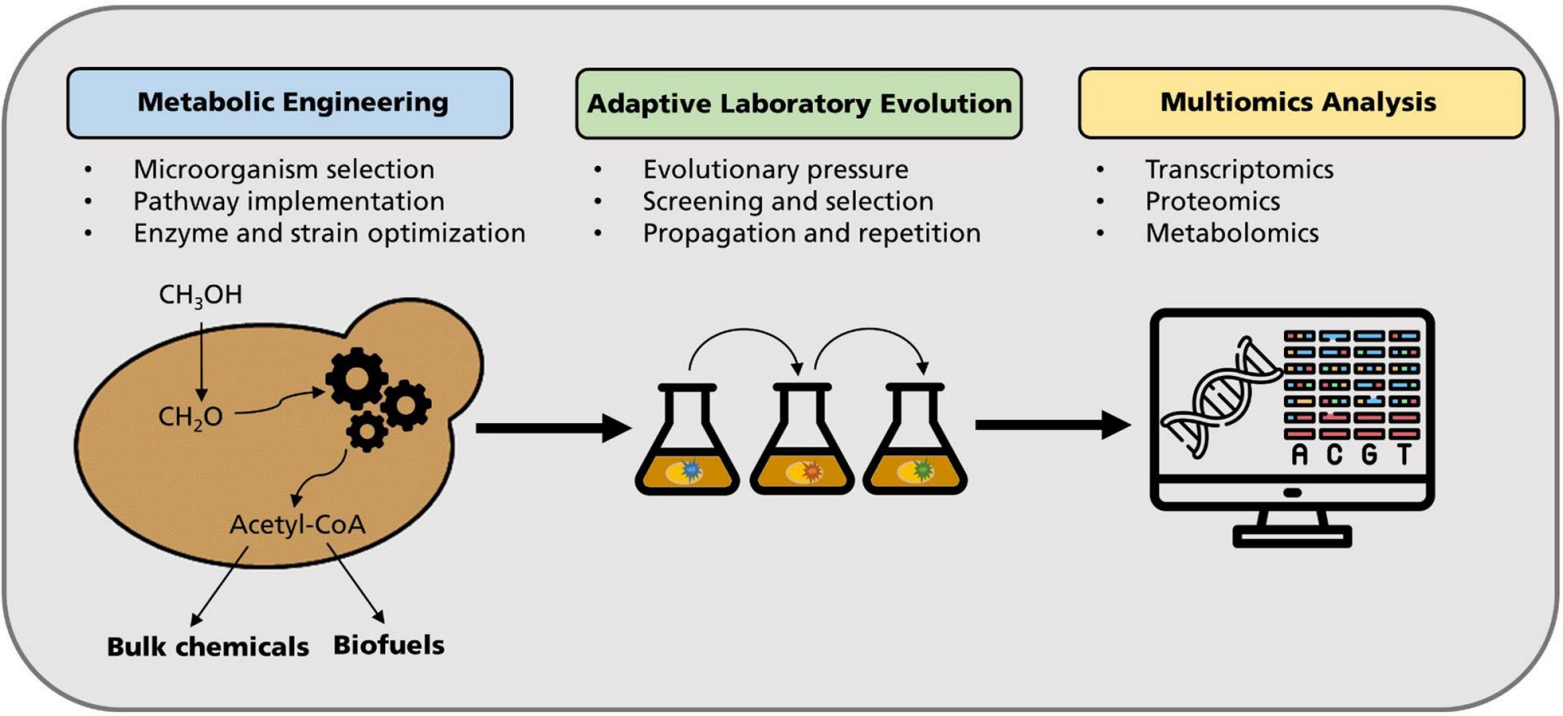
The road towards synthetic methylotrophic yeasts. The combination of metabolic engineering and adaptive laboratory evolution leads to exciting advances in the utilization of C_1_ compounds (e.g., methanol) as building blocks for the sustainable production of bulk chemicals and biofuels. Evolved strains are characterized for fitness improvements and integrated multiomics analysis helps to identify the most important changes on systems level, thus providing a deeper understanding of methylotrophy in general

The most recent findings suggest, that at this stage, ALE plays a more important role than rational metabolic engineering in constructing synthetic methylotrophy. By combining both strategies, exciting advances for using C_1_ compounds as a feedstock for synthetic methylotrophic eukaryotes can be reached (Fig. 4).

In conjunction with next-generation sequencing and omics-technologies, ALE can reveal relationships between genotypes and phenotypes, as well as the molecular mechanisms underlying the desired complex phenotypes. However, in order for ALE to be successful in generating strains with improved C_1_-utilization, the substrate-of-interest should be coupled with cellular growth or survival. A combination of advanced metabolic engineering, in silico modeling, and automation to maximize evolutionary efficiency should be considered. Finally, the subsequent omics-analysis of the evolved strains can lead to new insights into the mode of action and further genetic targets to improve efficiency of synthetic methylotrophy in eukaryotes even more [129].

### Recent advantages of formate utilization by yeasts

Besides methanol, formate is another attractive C_1_ compound, which can be generated in a renewable manner from electrochemical reduction of CO_2_ and used as a soluble feedstock [130]. In terms of aerobic growth, the synthetic reductive glycine pathway was identified as the most efficient route [131]. *S. cerevisiae* natively harbors NAD^+^-dependent FDH as well as all the enzymatic components needed for the reductive glycine pathway and could therefore serve as an ideal host. Via overexpression of these endogenous enzymes, glycine biosynthesis from formate and CO_2_ was achieved. Interestingly, growth rates of this engineered strain remained more or less constant for formate concentrations between 1 and 500 mM, indicating high tolerance as well as high affinity towards this substrate [132]. These findings raise the question why overexpression of the reductive glycine pathway was necessary to enable growth on formate in the first place. It is speculated that although formate is a common metabolic intermediate in eukaryotic cells, it is not usually present in the natural environment of this yeast. Therefore, the cells were unable to take it up efficiently [132]. Although, as of today, there is still no pure formatotrophic *Y. lipolytica* strain. However, an innovative fermentation process which cofeeds glucose and formate was developed. It was shown that co-feeding formate and glucose, up to a molar ratio of ~ 5:1, linearly increased the biomass yield of *Y. lipolytica*. Consistent with previous observations in other yeasts, it is hypothesized that consumption of formate under these conditions has a positive net ATP yield and therefore promotes growth [133]. While both examples are first steps towards the efficient utilization of formate in yeasts, there is still room to better exploit its potential.

To wrap it up, the on-going research shows that vast steps were made to unlock synthetic methylotrophy in various species including yeasts. However, many challenges and unknown aspects of metabolic traits, or effects of metabolic interventions when installing synthetic methylotrophy, remain unsolved. Especially the role of the pentose phosphate pathway and its interconnection with biosynthesis of complex metabolites or biomass associated molecules is of interest. Finally, the finding that methylotrophy is present in common glycolytic yeast strains has a striking potential to elucidate new engineering strategies for establishing efficient microbial cell factories for methanol-based production.

## Future directions

Due to the depletion of fossil fuels and concerns about environmental pollution, there is an urgent need to develop sustainable and climate neutral products and chemicals. In this regard, the application of CO_2_-derived C_1_ feedstock received great attention. One reason is that the key feedstock CO_2_, is virtually unlimited [134]. Therefore, start-ups and established companies strived into the field and research efforts are on-going. The valorization of gaseous C_1_ substrates might be one possibility in the solution space to face climate change and obtain sustainable commercial production processes for bulk- and fine-chemicals as well as biofuels. In addition, CCU approaches to fix CO_2_ into methanol or formate followed by fermentation is promising for future directions into a sustainable and cyclic bioeconomy.

The nature of methylotrophs empowers these microbes to utilize renewable derived C_1_ feedstock, depicting them as attractive biotechnological platform strains for industrial strain development. In particular, from the viewpoint of the bioprocess, these strains provide key features to establish sustainable bioprocesses. Nevertheless, challenges remain and limit their broad use on large and commercially feasible scales so far, in terms of applying gaseous or liquid substrates. In particular, remaining challenges are low conversion and growth rates using gaseous substrates, low biomass yields and a lack of reliable genetic engineering tools when considering native methylotrophs. From the viewpoint of synthetic methylotrophy, implementation of functional genetic methylotrophic modules in established industrial host organism were so far introduced but are still limited. To date, the literature indicates that the installation of metabolic regeneration cycles such as supporting carbon re-entry towards the pentose phosphate pathway from C_1_ fueled central carbon metabolism is a crucial target for synthetic methylotrophy. Moreover, the recent engineering of industrially relevant microbes, such as *E. coli* or *S. cerevisiae*, towards utilization of methanol or formate as the sole carbon source succeeded. Despite this, the future for both approaches, native and synthetic methylotrophy, seems promising, as the tools and technologies are now emerging to push the frontier towards efficient C_1_-utilization in a modern bioeconomy.
